# KRAS mutation analysis in ovarian samples using a high sensitivity biochip assay

**DOI:** 10.1186/1471-2407-9-111

**Published:** 2009-04-09

**Authors:** Veronika Auner, Gernot Kriegshäuser, Dan Tong, Reinhard Horvat, Alexander Reinthaller, Alexander Mustea, Robert Zeillinger

**Affiliations:** 1 Division of Gynaecology, Department of Obstetrics and Gynaecology, Medical University of Vienna, Vienna, Austria; 2 ViennaLab Diagnostics GmbH, Vienna, Austria; 3 Department of Clinical Pathology, Medical University of Vienna, Vienna, Austria; 4 Department of Obstetrics and Gynaecology, Charité Medical University Berlin Campus Virchow-Klinikum, D-13353 Berlin, Germany

## Abstract

**Background:**

Mutations in the *KRAS *gene are one of the most frequent genetic abnormalities in ovarian carcinoma. They are of renewed interest as new epidermal growth factor receptor (EGFR)-targeted therapies are being investigated for use in ovarian carcinoma. As *KRAS *mutations are associated with poor response and resistance to EGFR-targeting drugs, this study was conducted to obtain more information on the spectrum of *KRAS *mutations in ovarian carcinoma.

**Methods:**

The presence of *KRAS *mutations in codon 12 and 13 was analyzed in frozen and formalin-fixed paraffin-embedded (FFPE) tissue with a low density biochip platform. 381 malignant (29 borderline malignancy, 270 primary carcinomas, and 82 recurrent carcinomas) and 22 benign tissue samples from a total of 394 patients were examined. *KRAS *mutational status of each sample was correlated with dignity, FIGO stage, grade, histology, and survival.

**Results:**

*KRAS *mutations were found in 60 (15%) samples with 58 samples deriving from malignant tissue and 2 samples deriving from benign tissue. In 55 (92%) samples codon 12 was found to be mutated. Frozen and FFPE samples concurred with respect to *KRAS *mutation status.

**Conclusion:**

*KRAS *mutation is a common event in ovarian cancer primarily in carcinomas of lower grade, lower FIGO stage, and mucinous histotype. The *KRAS *mutational status is no prognostic factor for patients treated with standard therapy. However, in line with experience from colorectal cancer and non-small-cell-lung cancer (NSCLC), it may be important for prediction of response to EGFR-targeted therapies.

## Background

According to WHO statistics reported in 2005, ovarian cancer was ranked 5th in cancer related death in Europe.

As in other tumors, the triggers of ovarian cancer involve genetic alterations and mutations. The discovery of underlying mechanisms that lead to an adverse patient outcome is of great importance.

One of the best known DNA alterations in a variety of cancers is the mutational activation of ras protein family members. In fact, 20–30% of all human tumors harbour a mutation in a member of the ras family [1]. In ovarian cancer, *KRAS *mutations belong to the most frequently observed abnormalities [2].

The ras proteins are small GTPases, downstream of EGFR, which normally cycle between their active and inactive state. In health, ras proteins are key components in many pathways that couple growth factor receptors to downstream mitogenic effectors involved in cell proliferation and differentiation.

The most common mutations of these genes are found in codons 12, 13 and 61, which lead to a constitutively active ras protein, and subsequently to an inappropriate increase in proliferation and malignant transformation [1]. In ovarian cancer, mutations most often affect codons 12 and 13 of the *KRAS *gene, with mutation rates reported to be as high as 3–11% [3,4]. Mutations in codon 61 are rare in ovarian cancer [5,6], and therefore will not be considered in this study.

It is known that non-small-cell-lung cancer (NSCLC) and colorectal cancer (CRC) patients with mutant *KRAS *show poor response to anti-EGFR therapy [7-10]. Because ovarian cancer patients are also being considered for treatment with EGFR-targeting substances, and as clinical trials are already conducted, it is crucial to know whether patients harbouring a *ras *mutation may benefit from such therapy.

In this study, 403 malignant and benign samples from 394 patients were analyzed for the presence of mutations in the *KRAS *gene at codons 12 and 13 using a biochip array hybridization method called GeneStix [11]. To our knowledge, this is the largest number of ovarian cancer samples analyzed in this manner with the sample size being twice as large as in the studies of Sieben [12] and Gemignani [13].

## Methods

### Patients

403 samples from 394 patients were examined. 381 malignant tissue samples (borderline malignancies, primary and recurrent ovarian carcinomas) from 377 patients and 22 samples from benign tissue (6 ovarian tissues and 16 ovarian cystadenomas) derived from 21 patients were analyzed. More than one sample was available for a total of 9 patients; including primary and recurrent lesions for 3 patients, 2 distinct recurrent lesions for 1 patient, healthy tissue and primary lesions for 2 patients, healthy and borderline tissue for 1 patient, and cystadenoma and healthy tissue for 2 patients.

170 samples were obtained from patients treated at the Department of Obstetrics and Gynaecology, Charité University Hospital, Campus Virchow, Berlin. 233 samples were collected at the Department of Obstetrics and Gynaecology, Medical University Vienna, Vienna. Samples were collected between 1989 and 2006.

Tissue attained in Berlin was fresh frozen to -80°C, and samples from Vienna were either formalin-fixed paraffin-embedded (FFPE) (n = 110) or fresh frozen to -80°C (n = 123). Patients gave their written informed consent and the study was approved by the local institutional review boards.

### Preparation of Samples

FFPE samples were punched out of tissue blocks (Tissue Microarrayer I; Beecher Instruments, Sun Prairie, USA) and the DNA was extracted according to Fabjani et al. [14].

Briefly, the samples were washed repeatedly with xylene and ethanol and thereafter dried. Each dried pellet was resuspended in 250 μL of a solution containing 12.5 mg glass beads (Glass Controlled Pore, 120–200 μm mesh; Sigma), PCR amplification-buffer, and Proteinase K (0.6 mg/mL). The samples were incubated in an ultrasonic water bath at 56°C for 30 min followed by incubation at 95°C 10 min in a conventional thermoblock (Eppendorf AG, Hamburg, Germany) to inactivate proteinase K. Subsequently, the samples were centrifuged and the DNA containing supernatant was used for further analysis.

DNA from frozen samples obtained in Vienna was isolated using a salt elution method. Briefly, up to 1 g tissue was pulverized with a microdismembrator. The powdered tissue was suspended in 3 mL lysis buffer (2.34% NaCl, 10 mM Tris-HCl and 1 mM EDTA, pH = 8). 100 μL solubilizer (20% sodium dodecyl sulfate solution) and 100 μL of 2% proteinase K (Sigma-Aldrich, St. Louis, USA) solution were added. Lysis was performed by overnight incubation at 37°C with gentle agitation. After cooling the sample to room temperature (RT), 1.4 mL saturated salt solution was added. The mixture was centrifuged at 10000 × g for 15 min at RT and DNA was precipitated by adding 2 volumes of 100% ethanol and the supernatant removed. After air-drying for 5 minutes, DNA was dissolved in 200 μL Tris-EDTA buffer (10 mM Tris-HCl, 1 mM EDTA, pH = 8).

The DNeasy Blood & Tissue Kit (Qiagen, Hilden, Germany) was used to isolate DNA from samples collected in Berlin.

### Mutation Analysis

The GeneStix biochip platform has been optimized for the simultaneous detection of 10 frequent mutations found in codons 12 and 13 of the *KRAS *gene [11].

The biochip consists of a plastic stick with DNA capture oligonucleotides immobilized at the tip, and the entirely contained in a cylindrical tube. Samples were analyzed by hybridization to the biochip array after mutant-enriched PCR as described by Fabjani et al. [15].

In summary, downstream primers were biotin-labeled, upstream primers were phosphorylated at the 5' position. 20 μL of the PCR products were digested with 1 μL λ-Exonuclease (New England BioLabs, Ipswich, USA) for 30 minutes at RT. In a GeneStix tube, 10 μL of the digested PCR products were diluted in 200 μL hybridization buffer including a hybridization control target. Stick hybridization was performed at 37°C for 1 h in a conventional thermoshaker (Eppendorf AG, Hamburg, Germany).

Without additional washing steps, the biochip was stained for 15 min with the provided streptavidin-horseradish peroxidase conjugate, and thereafter rinsed with 1 mL of assay buffer. The stick was then analyzed with the GeneStix-Imager, a chemiluminescence detector developed for use with the GeneStix system. Images were automatically processed with the test-specific analysis software provided with the imager.

Previously, the assay's limit for detecting *KRAS *mutations was determined using 0.1 ng of tumor cell line DNA mixed with 100 ng of wild-type DNA [11]. Even in this 1000-fold excess of wild-type DNA, GeneStix hybridization unambiguously identified all *KRAS *mutations covered by the assay. In the same study, 26 stool samples were analyzed in parallel by biochip hybridization and sequencing. Biochip-based hybridization detected *KRAS *mutations in 9/26 (35%) samples, all of which were confirmed by sequencing.

### Statistics

To test for differences between groups, in regard to mutation rate, the Pearson's chi-square test was used. Tests included mutational rates of borderline lesions versus carcinoma, primary versus recurrent carcinoma, grade 1 mucinous tumors versus higher grade mucinous tumors, and the incidence of recurrence in primary carcinoma.

For deeper insight into factors consisting of more than two groups, such as overall and mucinous histotype grading (1–3), FIGO stage (I–III), and nodal status in carcinoma, and for histology (serous, mucinous, endometrioid, others/unknown) in all malignant samples, a residual analysis with adjusted standardized residuals was done.

To test for relation between patient age and the mutational rate, a Mann-Whitney-U test was performed.

For patients with primary ovarian cancer overall and recurrence free survival was calculated with univariate Log rank and Cox regression adjusted for FIGO. All statistical calculations were conducted with SPSS 15.0 (SPSS Inc; Chicago, IL, USA).

## Results

### Basic results

Of the 403 samples examined, 60 (15%) contained mutations at codon 12 or 13. The most frequent mutation was asp12 (40%) followed by val12 (23%) and cys12 (13%). Overall, 92% of the mutations were located in codon 12. Two patients carried double mutations (Table 1).

**Table 1:** KRAS mutations examined samples

mutation	amino acid	n	%
GGT → GAT	gly12 → asp12	24	40

GGT → TGT	gly12 → cys12	8	13

GGT → GTT	gly12 → val12	14	23

GGT → GCT	gly12 → ala12	4	7

GGT → CGT	gly12 → arg12	2	3

GGC → GAC	gly13 → asp13	4	7

GGC → TGC	gly13 → cys13	1	2

GGT → GCT/GAT	gly12 → ala12/asp12	2	3

GGT → GTT/GAT	gly12 → val12/asp12	1	2

	total	60	100

Overall, mutations were more common in borderline lesions (35%) than in primary carcinoma (16%) and recurrent ovarian carcinoma (6%). Two of the 16 ovarian cystadenoma samples contained mutations, no mutation was found in healthy ovarian tissue.

Multiple samples were analyzed from 9 patients. A *KRAS *mutation was detected in only one of these. Of this patient, the primary and recurrent lesion could be analyzed and identical mutations were found in both lesions.

Mucinous lesions displayed mutations most frequently. The mutational rate increased from 50% in mucinous borderline lesions to 60% in primary tumors of the same histotype (Tables 2 and 3). In serous lesions *KRAS *mutations occurred less frequently. Compared to mucinous lesions, serous malignancies exhibited a relatively high mutational frequency in borderline tumors (35%) and fewer mutations in carcinoma (9%) (Tables 2 and 3).

**Table 2:** KRAS mutation frequencies observed in borderline malignancies

	borderline		
	
histotype	n	mutated	% mutated
serous	20	7	35.00

endometroid	1	0	0.00

mucinous	6	3	50.00

unknown	2	0	0.00

total	29	10	34.48

**Table 3:** KRAS mutation frequencies observed in carcinoma

	primary carcinoma			recurrent carcinoma			carcinoma total		
	
	n	mutated	% mutated	n	mutated	% mutated	n	mutated	% mutated
	270	43	15.93	82	5	6.10	352	48	13.64

**histotype**									

serous	177	17	9.60	69	4	5.80	246	21	8.54

endometroid	34	4	11.76	5	0	0.00	39	4	10.26

mucinous	25	15	60.00	5	1	20.00	30	16	53.33

clear cell	19	5	26.32	2	0	0.00	21	5	23.81

undifferent.	10	0	0.00	1	0	0.00	11	0	0.00

unknown	5	2	40.00				5	2	40.00

									

**age**									

≤ 50	72	16	22.22	31	4	12.90	103	20	19.42

> 50	198	27	13.64	51	1	1.96	249	28	11.24

									

**grading**									

1	33	17	51.52	7	0	0.00	40	17	42.50

2	90	14	15.56	27	4	14.81	117	18	15.38

3	141	12	8.51	46	1	2.17	187	13	6.95

4	1	0	0.00	0	0	0.00	1	0	0.00

unknown	5	0	0.00	2	0	0.00	7	0	0.00

									

**FIGO stage**									

1	65	17	26.15	9	0	0.00	74	17	22.97

2	31	5	16.13	8	0	0.00	39	5	12.82

3	145	17	11.72	48	4	8.33	193	21	10.88

4	28	4	14.29	13	1	7.69	41	5	12.20

unknown	1	0	0.00	4	0	0.00	5	0	0.00

									

**pN**									

0	104	17	16.35	19	0	0.00	123	17	13.82

1	67	7	10.45	28	4	14.29	95	11	11.58

x	99	19	19.19	35	1	2.86	134	20	14.93

Data from primary carcinoma was split up into FFPE and fresh frozen samples. Overall the percentage of mutation was very similar (14% in FFPE tissue vs. 16% in fresh frozen tissue). Divided according to histotype the results were comparable. The only exception was seen in the mutational rate of mucinous tumors. This rate was much higher in FFPE samples. This is probably an artefact caused by the small sample size for this particular histotype (Table 4).

**Table 4:** KRAS mutations in FFPE tissue and frozen tissue

	FFPE tissue			frozen tissue		
	
histotype	n	mutated	% mutated	n	mutated	% mutated
serous	50	2	4,00	127	15	11,81

mucinous	9	8	88,89	16	7	43,75

others	29	3	10,34	39	8	20,51

total	88	13	14,77	182	30	16,48

### Statistical results

Pearson's chi-square test was used to compare mutation rates of borderline to cancerous lesions and primary to recurrent disease.

Borderline lesions more frequently contained mutations than tumors (p = 0.003), and the rate of mutation was significantly higher in primary tumors than recurrent tumors (p = 0.023).

To get more insight into the relation between the mutation rate of carcinoma, nodal status, tumor grade (1–3), and FIGO classification (I–III) residual analysis was used.

Tumors with different grading showed a significant difference in mutation frequency.

Grade 1 tumors exhibited a significantly higher mutational rate than grade 3 tumors (p < 0.0001). We found no significant difference in mutational rates in patients with different FIGO stages, but a trend towards a higher mutational rate could be seen in FIGO stage I tumors (p = 0,081). No differences were found in rates of mutation related to nodal status.

When malignant samples were compared with respect to histology, the mutational rate in mucinous lesions was significantly higher than in lesions of other histotypes, whereas serous malignancies harboured significantly less *KRAS *mutations (p < 0.0001).

Because *KRAS *mutations are a typical feature of mucinous lesions, we studied the results of this subtype more closely. Similar to the statistical results independent of histotype, the mutation rates are significant higher in grade 1 carcinoma compared to higher grade mucinous tumors (p = 0,007).

We also found, that tumors originating from younger patients more frequently contained a *KRAS *mutation (Mann-Whitney-U, p = 0.063). No statistical difference was found between the groups of patients with and without recurrence.

Furthermore, no significant difference could be found in recurrence free and overall survival, between patients with mutated and wildtype *KRAS *tumors. The log-rank test resulted in p-values of 0.258 for recurrence free, and 0.567 for overall survival respectively. FIGO corrected Cox regression showed p-values of 0.865 and 0.656 for recurrence free and overall survival.

## Discussion

As of late *KRAS *mutations in ovarian cancer should stand in the center of attention again due to the availability of EGFR-targeted therapies (e.g. Gefitinib, Erlotinib, and Cetuximab), that are already in use to treat patients with NSCLC and metastatic CRC. These therapies are under discussion for treatment of ovarian cancer and numerous clinical trials are already in progress [16], however, not all patients respond equally to this treatment. In patients with NSCLC and CRC mutations in *EGFR *lead to a better prognosis under this medication [17-19], others show marginal if any response. Recently, *KRAS *activational mutations have been associated with a lack of sensitivity to anti-EGFR substances by maintaining the ability to continue aberrant signalling through the EGFR pathway, circumventing the blocked EGFR [7-10].

It has been shown that EGFR mutations do not play a role in ovarian cancer [20], while *KRAS *mutations are very well documented [3,4,6,21].

The frequency of *KRAS *mutations was similar in frozen and FFPE tissue, indicating that our method is suitable for *KRAS *analysis in both types of tissue.

The mutation frequency found in borderline malignancies (34%) and carcinomas (13%), is well in line with previous reports [3-5,22]. Not surprisingly, benign ovarian tissue contained a minimal number of mutations.

Overall, the mutation rate in mucinous malignancies is much higher than in lesions of any other histological subtype. It is likely that *KRAS *mutation is associated with mucinous differentiation [23] as these mutations also accumulate in mucinous carcinomas obtained from other organs [24]. In agreement with other studies, a lower mutation rate was found in mucinous borderline malignancies compared to carcinoma [4,24].

In line with other studies, we also find a smaller percentage of samples harbouring a mutation in serous borderline tumors than in serous carcinoma. In serous lesions *KRAS *mutation seems to be a typical feature of borderline tumors and low grade carcinoma. On the other hand, *KRAS *mutation does not seem to play a major role in high grade serous carcinoma. Russel and McCluggage [25] and also Kurman and Shih [21] proposed a new theory that distinguishes between high grade and low grade serous carcinoma and tries to reconstruct the different steps of development in those entities. *KRAS*, *BRAF *and *PTEN *mutations seem to be a feature of tumors with low malignant potential, whereas high grade carcinoma, which account for 90% of all serous carcinoma, harbour *TP53 *mutations and genetic instability [21]. The results of our study may not supply a new foundation for this theory; however, it stands well in line with the established notion of two separate pathways for the development of high and low grade serous ovarian carcinoma.

When *KRAS *mutational status was analyzed irrespective of histotype, grade 1 tumors showed a significantly higher mutational rate than grade 3 tumors. This is probably due to the predominance of serous samples in the group of high grade tumors. An almost equal number of serous and mucinous lesions are present in the group of grade 1 tumors. The group of grade 2 tumors contain 5 times more, and the group of grade 3 tumors over 50 times more serous than mucinous lesions. As stated above, serous tumors seldom harbour a *KRAS *mutation. Even if a mutation occurs, it will appear more probable in serous lesions with low malignant potential which would even amplify the described effect.

The higher number of *KRAS *mutations in lower grade samples could also be an explanation for the higher mutational rate in younger patients. Various studies have shown that patients with lower grading seem to be of younger age than patients with high grading [21].

Mutant and wildtype primary carcinoma showed no significant difference regarding the incidence of recurrent disease (p = 0.114). There is also no difference in recurrence free survival or overall survival. Therefore, *KRAS *mutation cannot be considered a prognostic factor for standard chemotherapy.

The percentage of analyzed recurrent tumors bearing a mutation was therefore surprisingly low. The imbalance may be explained by the relatively small sample size of recurrent carcinomas and may be lost in the large sample variation.

One of the patients for whom multiple samples were analyzed, showed a mutation in the *KRAS *gene. As this mutation was found in both the primary carcinoma and the recurrent lesion, the mutational event must have taken place in the primary lesion previous to the processes that led to recurrent disease.

Diebold et al. report six cases where tissue was taken from two contralateral borderline tumor masses harbouring a *KRAS *mutation. In all cases the mutations were present in each of the paired tumors examined [26]. Ho et al. detected corresponding mutations in borderline malignancies and the adjacent cystadenoma epithelium [27]. Therefore it could be concluded that mutations of *KRAS *are an early event in development of low grade tumors.

Our finding of one paired mutated primary and recurrent tumor is hardly enough evidence, but supports earlier findings from other groups and further supports the notion that such mutations are an early event in the malignant process.

## Conclusion

In summary, *KRAS *mutation is a common event in ovarian cancer and is more frequently present in carcinoma of lower grade, lower FIGO stage, and in lesions of mucinous histotype. Our results support earlier findings from other groups in a very large number of samples. *KRAS *mutation was not found to be of prognostic value for patients under standard therapy, but these mutations could emerge as an important factor for individually tailored anti-EGFR therapies.

## Competing interests

The authors declare that they have no competing interests.

## Authors' contributions

VA and GK contributed equally to this project. Both did the practical work, wrote the text and did statistical analysis. RH was in charge of the analysis of the histological data from Viennese samples AR selected and provided clinical samples from Vienna, AM was responsible for acquisition and selection of the clinical samples from Berlin. RZ and DT designed and supervised the project and emended the manuscript. All authors read and approved the final manuscript.

## Pre-publication history

The pre-publication history for this paper can be accessed here:

http://www.biomedcentral.com/1471-2407/9/111/prepub

## References

[B1] Bos JL ras oncogenes in human cancer: a review Cancer Res 1989 49 17 4682 4689 2547513

[B2] Nakayama N Nakayama K Yeasmin S Ishibashi M Katagiri A Iida K Fukumoto M Miyazaki K KRAS or BRAF mutation status is a useful predictor of sensitivity to MEK inhibition in ovarian cancer Br J Cancer 2008 99 12 2020 2028 10.1038/sj.bjc.6604783 19018267 PMC2607229

[B3] Mayr D Hirschmann A Lohrs U Diebold J KRAS and BRAF mutations in ovarian tumors: a comprehensive study of invasive carcinomas, borderline tumors and extraovarian implants Gynecol Oncol 2006 103 3 883 887 10.1016/j.ygyno.2006.05.029 16806438

[B4] Caduff RF Svoboda-Newman SM Ferguson AW Johnston CM Frank TS Comparison of mutations of Ki-RAS and p53 immunoreactivity in borderline and malignant epithelial ovarian tumors Am J Surg Pathol 1999 23 3 323 328 10.1097/00000478-199903000-00012 10078924

[B5] Enomoto T Weghorst CM Inoue M Tanizawa O Rice JM K-ras activation occurs frequently in mucinous adenocarcinomas and rarely in other common epithelial tumors of the human ovary Am J Pathol 1991 139 4 777 785 1656759 PMC1886299

[B6] Teneriello MG Ebina M Linnoila RI Henry M Nash JD Park RC Birrer MJ p53 and Ki-ras gene mutations in epithelial ovarian neoplasms Cancer Res 1993 53 13 3103 3108 8319218

[B7] Lievre A Bachet JB Boige V Cayre A Le Corre D Buc E Ychou M Bouche O Landi B Louvet C KRAS mutations as an independent prognostic factor in patients with advanced colorectal cancer treated with cetuximab J Clin Oncol 2008 26 3 374 379 10.1200/JCO.2007.12.5906 18202412

[B8] Khambata-Ford S Garrett CR Meropol NJ Basik M Harbison CT Wu S Wong TW Huang X Takimoto CH Godwin AK Expression of epiregulin and amphiregulin and K-ras mutation status predict disease control in metastatic colorectal cancer patients treated with cetuximab J Clin Oncol 2007 25 22 3230 3237 10.1200/JCO.2006.10.5437 17664471

[B9] Eberhard DA Johnson BE Amler LC Goddard AD Heldens SL Herbst RS Ince WL Janne PA Januario T Johnson DH Mutations in the epidermal growth factor receptor and in KRAS are predictive and prognostic indicators in patients with non-small-cell lung cancer treated with chemotherapy alone and in combination with erlotinib J Clin Oncol 2005 23 25 5900 5909 10.1200/JCO.2005.02.857 16043828

[B10] van Zandwijk N Mathy A Boerrigter L Ruijter H Tielen I de Jong D Baas P Burgers S Nederlof P EGFR and KRAS mutations as criteria for treatment with tyrosine kinase inhibitors: retro- and prospective observations in non-small-cell lung cancer Ann Oncol 2007 18 1 99 103 10.1093/annonc/mdl323 17060486

[B11] Prix L Uciechowski P Bockmann B Giesing M Schuetz AJ Diagnostic biochip array for fast and sensitive detection of K-ras mutations in stool Clin Chem 2002 48 3 428 435 11861435

[B12] Sieben NL Macropoulos P Roemen GM Kolkman-Uljee SM Jan Fleuren G Houmadi R Diss T Warren B Al Adnani M De Goeij AP In ovarian neoplasms, BRAF, but not KRAS, mutations are restricted to low-grade serous tumours J Pathol 2004 202 3 336 340 10.1002/path.1521 14991899

[B13] Gemignani ML Schlaerth AC Bogomolniy F Barakat RR Lin O Soslow R Venkatraman E Boyd J Role of KRAS and BRAF gene mutations in mucinous ovarian carcinoma Gynecol Oncol 2003 90 2 378 381 10.1016/S0090-8258(03)00264-6 12893203

[B14] Fabjani G Kucera E Schuster E Minai-Pour M Czerwenka K Sliutz G Leodolter S Reiner A Zeillinger R Genetic alterations in endometrial hyperplasia and cancer Cancer Lett 2002 175 2 205 211 10.1016/S0304-3835(01)00714-5 11741749

[B15] Fabjani G Kriegshaeuser G Schuetz A Prix L Zeillinger R Biochip for K-ras mutation screening in ovarian cancer Clin Chem 2005 51 4 784 787 10.1373/clinchem.2004.041194 15788786

[B16] Palayekar MJ Herzog TJ The emerging role of epidermal growth factor receptor inhibitors in ovarian cancer Int J Gynecol Cancer 2008 18 5 879 890 10.1111/j.1525-1438.2007.01144.x 18053062

[B17] Zhu CQ da Cunha Santos G Ding K Sakurada A Cutz JC Liu N Zhang T Marrano P Whitehead M Squire JA Role of KRAS and EGFR as biomarkers of response to erlotinib in National Cancer Institute of Canada Clinical Trials Group Study BR.21 J Clin Oncol 2008 26 26 4268 4275 10.1200/JCO.2007.14.8924 18626007

[B18] Endo K Sasaki H Yano M Kobayashi Y Yukiue H Haneda H Suzuki E Kawano O Fujii Y Evaluation of the epidermal growth factor receptor gene mutation and copy number in non-small cell lung cancer with gefitinib therapy Oncol Rep 2006 16 3 533 541 16865253

[B19] Karapetis CS Khambata-Ford S Jonker DJ O'Callaghan CJ Tu D Tebbutt NC Simes RJ Chalchal H Shapiro JD Robitaille S K-ras mutations and benefit from cetuximab in advanced colorectal cancer N Engl J Med 2008 359 17 1757 1765 10.1056/NEJMoa0804385 18946061

[B20] Mustea A Sehouli J Fabjani G Koensgen D Mobus V Braicu EI Pirvulescu C Thomas A Tong D Zeillinger R Epidermal growth factor receptor (EGFR) mutation does not correlate with platinum resistance in ovarian carcinoma. Results of a prospective pilot study Anticancer Res 2007 27 3B 1527 1530 17595771

[B21] Kurman RJ Shih Ie M Pathogenesis of ovarian cancer: lessons from morphology and molecular biology and their clinical implications Int J Gynecol Pathol 2008 27 2 151 160 18317228 10.1097/PGP.0b013e318161e4f5 PMC2794425

[B22] Schuyer M Henzen-Logmans SC Burg ME van der Fieret JH Derksen C Look MP Meijer-van Gelder ME Klijn JG Foekens JA Berns EM Genetic alterations in ovarian borderline tumours and ovarian carcinomas Eur J Obstet Gynecol Reprod Biol 1999 82 2 147 150 10.1016/S0301-2115(98)00217-6 10206406

[B23] Fujita M Enomoto T Murata Y Genetic alterations in ovarian carcinoma: with specific reference to histological subtypes Mol Cell Endocrinol 2003 202 1–2 97 99 12770737 10.1016/s0303-7207(03)00069-8

[B24] Cuatrecasas M Villanueva A Matias-Guiu X Prat J K-ras mutations in mucinous ovarian tumors: a clinicopathologic and molecular study of 95 cases Cancer 1997 79 8 1581 1586 10.1002/(SICI)1097-0142(19970415)79:8<1581::AID-CNCR21>3.0.CO;2-T 9118042

[B25] Russell SE McCluggage WG A multistep model for ovarian tumorigenesis: the value of mutation analysis in the KRAS and BRAF genes J Pathol 2004 203 2 617 619 10.1002/path.1563 15141374

[B26] Diebold J Seemuller F Lohrs U K-RAS mutations in ovarian and extraovarian lesions of serous tumors of borderline malignancy Lab Invest 2003 83 2 251 258 12594239 10.1097/01.lab.0000056994.81259.32

[B27] Ho CL Kurman RJ Dehari R Wang TL Shih Ie M Mutations of BRAF and KRAS precede the development of ovarian serous borderline tumors Cancer Res 2004 64 19 6915 6918 10.1158/0008-5472.CAN-04-2067 15466181

